# Supplementary figures and images for: Single cell transcriptome signatures and cell-cell interactions associated with sarcoidosis in lung immune cell populations
Source: Front Immunol. 2026 May 26;17:1765314. doi: 10.3389/fimmu.2026.1765314 (PMC13246344; doi:10.3389/fimmu.2026.1765314)

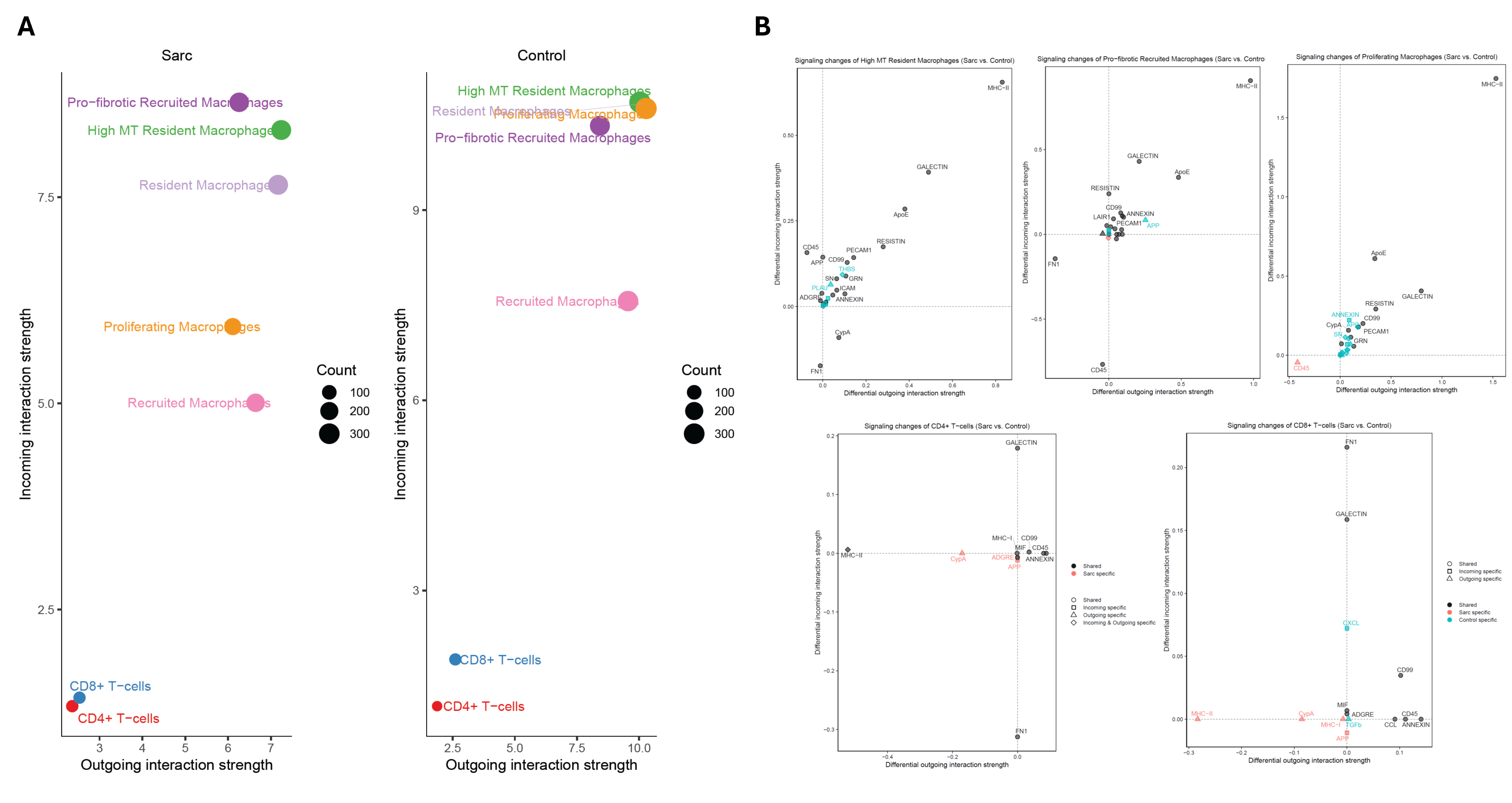

Supplement: Supplementary Figure 1 — (A) Scatterplots of cell populations with significant changes in sending or receiving signals. (B) Scatterplots of differential incoming and outgoing interaction strength for sarcoidosis compared to controls in five cell population (high MT resident macrophages, profibrotic recruited macrophages, proliferating macrophages, CD4+ and CD8+ T cells). [file Image1.tif]

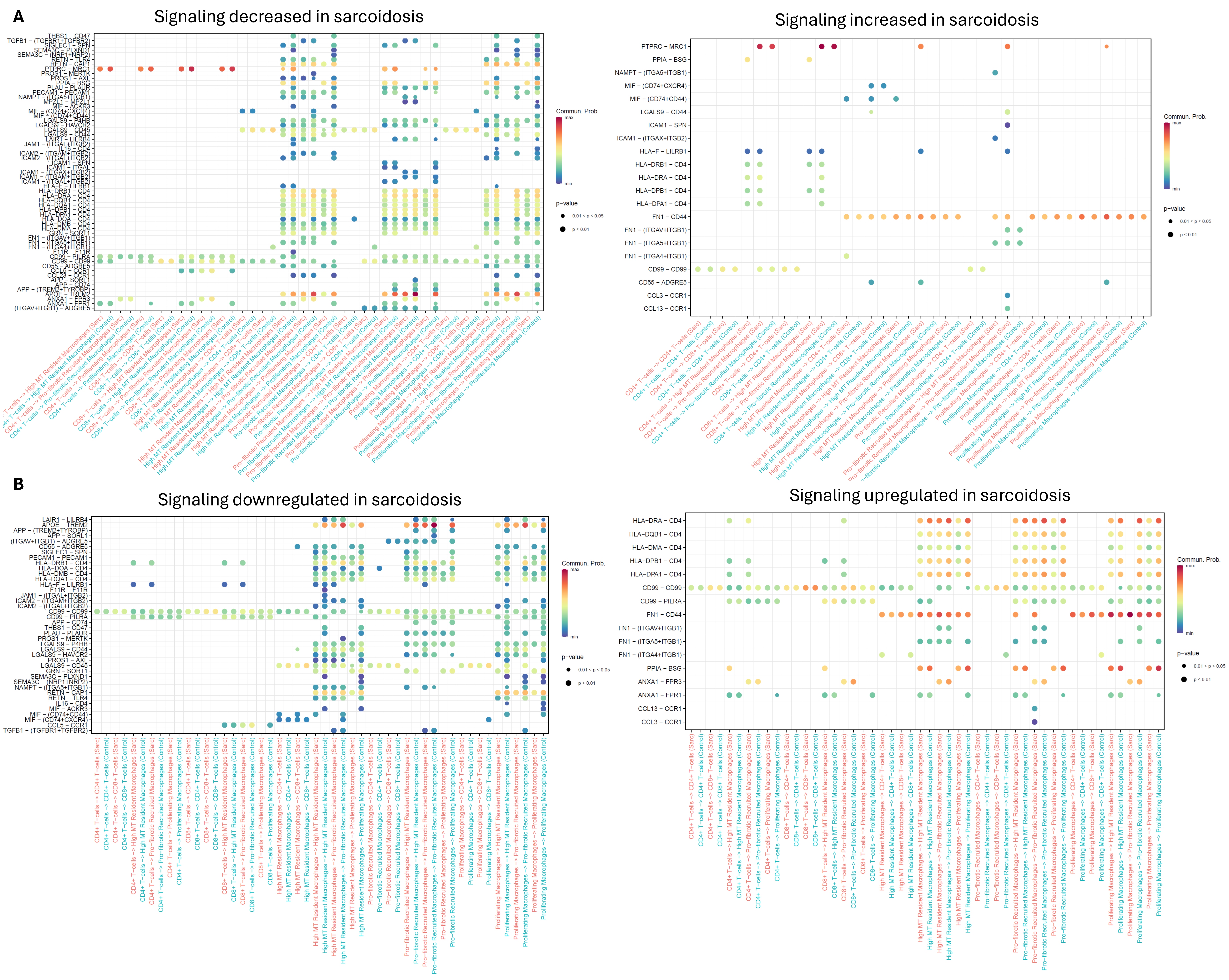

Supplement: Supplementary Figure 2 — Comparison of specific ligand-receptor interactions in specific cell types that are (A) increased or decreased in sarcoidosis, and (B) down- and up-regulated in sarcoidosis. [file Image2.tif]

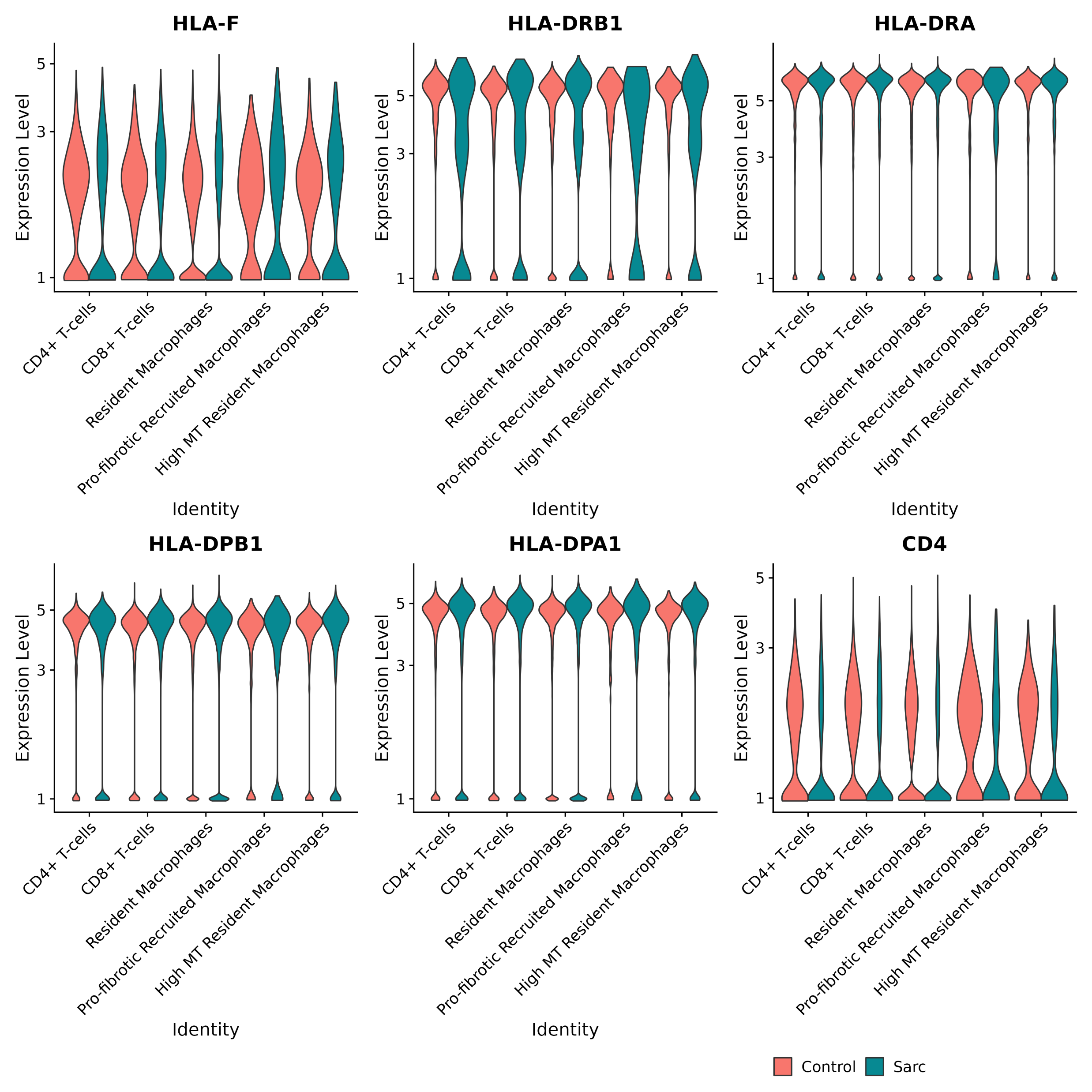

Supplement: Supplementary Figure 3 — Violin plots of select transcripts identified by CellChat interaction analysis between macrophage and T cell populations. [file Image3.tif]
